# External therapy of Chinese medicine for postherpetic neuralgia: A protocol for systematic review and meta-analysis

**DOI:** 10.1097/MD.0000000000023270

**Published:** 2020-12-11

**Authors:** Zheyi Wang, Yize Sun, Biyuan Liu, Zhu Fan, Jinzhou Tian, Tao Lu

**Affiliations:** a Beijing University of Chinese Medicine; b School of Life Science; c Dongzhimen Hospital, Beijing University of Chinese Medicine; d Guang’anmen Hospital, China Academy of Chinese Medical Sciences, Beijing, China.

**Keywords:** external therapy of Chinese medicine, meta-analysis, postherpetic neuralgia, protocol, systematic review

## Abstract

**Background:**

Postherpetic neuralgia (PHN), the most common complication of herpes zoster, brings about a health-care burden at both the individual and societal levels. External therapy of Chinese medicine (ETCM) is an effective treatment of PHN generally available in China, yet there is incomplete evidence to evaluate the efficacy and safety of it.

**Methods:**

This protocol is based on the previous reporting items. We will search 3 English databases (PubMed, EMBASE, and the Cochrane Library) and 3 Chinese databases (CNKI, CBM, and Wan Fang Database) until January 2020. RCTs to evaluate the efficacy and safety of external therapy of Chinese medicine for postherpetic neuralgia will be included. The primary outcome will be assessed by VAS or NRS. We will use the criteria provided by Cochrane Handbook 5.3.0 for quality evaluation and risk assessment, and use the Revman 5.3 software for meta-analysis.

**Ethics and dissemination:**

Ethical approval is not required for systematic review and meta- analysis. The results of this review will be disseminated in a peer-review journal.

**PROSPERO registration number:**

CRD42020163511.

## 1 Introduction

Herpes zoster is an infectious skin disease caused by reactivation of varicella zoster virus, which has long been latent in the posterior root ganglion or cranial ganglion of the spinal cord. The annual incidence of shingles is 3 to 5 per 1000 person-years^[1,2]^ and approximately a fifth of patients with it report some pain at 3 months after the onset of symptoms, and 15% report pain at 2 years.^[3]^ The most common complication of herpes zoster, postherpetic neuralgia (PHN), was defined as persistent pain for ≥3 months after the resolution of cutaneous lesions, the prevalence of which rises with age.^[3,4]^ It brings about a health-care burden at both the individual and societal levels, and patients with it suffer from reduced quality of life, physical functioning, and psychological well-being.^[5,6]^ The mechanisms of PHN mainly involve inflammatory response and disturbances within the central and peripheral nervous systems that induce an abnormal reorganization of the pain stimuli transmission system and a disorganized innervation pattern, thus creating the spontaneous pain.^[7,8]^ But the detail of the mechanism is ambiguous, making it difficult to advance the precise treatment of PHN.

In clinical trials, currently available therapies have a narrow therapeutic index and fewer than half of patients with postherpetic neuralgia have a 50% or greater reduction in pain^[3,9]^; adverse effects of oral medications are common (usually presenting with dizziness and drowsiness), particularly in older patients.^[10,11]^ Under the circumstances, multimodality therapy, for instance, a combination of both topical and systematic agents, is required for optimal outcomes.^[12,13]^ However, clinical evidence from randomized trials comparing combination topical and systemic therapy with either therapy alone is still limited.^[3,13]^ Furthermore, topical therapy may cause pruritus, erythema, and dermatitis when applied.^[14,15]^ In view of the above, novel and effective external strategies with much fewer side effects need to be developed.

In China, especially in community clinics and traditional Chinese medicine hospitals, external therapy of Chinese medicine (ETCM) has become a significant treatment for PHN, yet there is incomplete evidence to assess the efficacy of it.^[16,17]^ Therefore, we will make a systematic review and meta-analysis of published RCTs to evaluate the effectiveness and safety of ETCM, in order to provide more options for clinical external use.

## 2 Methods

### 2.1 Study registration

This systematic review protocol has been registered in PROSPERO (registration number: CRD42020163511). This protocol has been checked with preferred reporting items for systematic review and meta-analysis protocols (PRISMA-P) checklist.^[18]^

### 2.2 Inclusion criteria

#### 2.2.1 Type of studies

Only RCTs to evaluate the efficacy and safety of ETCM for PHN will be included. There is no language restriction on study selection.

#### 2.2.2 Participants

According to “consensus of experts on the diagnosis and treatment of post-herpetic neuralgia in China”,^[4]^ participants who are clinically diagnosed with PHN will be included. No restrictions on age, gender and race.

#### 2.2.3 Interventions

Treatment group employs external application of Chinese herbal medicine, including tincture, ointment, gelatin, pulvis, lozenge, transdermal stick, fumigation, lotion, and liniment, in combination with control group treatments, while control group uses routine therapies alone, or with external placebo. Routine therapies cover pregabalin, gabapentin, amitriptyline, and other oral medicine recommended by consensus, excluding topical therapy, acupuncture, and neurointervention.

#### 2.2.4 Outcomes

##### 2.2.4.1 The primary outcome

The primary outcomes are mainly evaluated by visual analogue scale (VAS) or numeric rating scale (NRS).

##### 2.2.4.2 The secondary outcome

The secondary outcomes are assessed by health status questionnaire (SF-36), pain time, and adverse effects.

### 2.3 Search strategy

#### 2.3.1 Database search

We will search for literatures until January 2020 from the following 6 databases: PubMed, Excerpta Medica Database (EMBASE), the Cochrane Library, China National Knowledge Infrastructure (CNKI), Wan Fang database, and Chinese Biomedical (CBM).

#### 2.3.2 Searching other resources

Meanwhile, we will search Chinese Clinical Trial Registry and the US National Institutes of Health Ongoing Trials Register for any related ongoing or unpublished trials.

#### 2.3.3 Search strategy

Details of search strategy are stated as follow:

#1 Search (post-herpetic neuralgia [Title/Abstract]) OR (postherpetic neuralgia [Title/Abstract])

#2 Search (tincture [Title/Abstract]) OR (ointment [Title/Abstract]) OR (gelatin [Title/Abstract]) OR (pulvis [Title/Abstract]) OR (lozenge [Title/Abstract]) OR (transdermal stick [Title/Abstract]) OR (fumigation [Title/Abstract]) OR (lotion [Title/Abstract]) OR (liniment [Title/Abstract])

#3 Search (traditional Chinese medicine [MeSH Terms]) OR (TCM [Title/Abstract]) OR (Chinese medicine [Title/Abstract]) OR (Chinese herbs [Title/Abstract])

#4 Search (“randomized, controlled trial” [MeSH Terms]) OR (“randomized controlled trial” [Title/Abstract]) OR (“clinical study” [Title/Abstract]) OR (“clinical trial” [Title/Abstract])

#1 AND #2 AND #3 AND #4

### 2.4 Data collection and analysis

#### 2.4.1 Study selection

**Figure 1:**
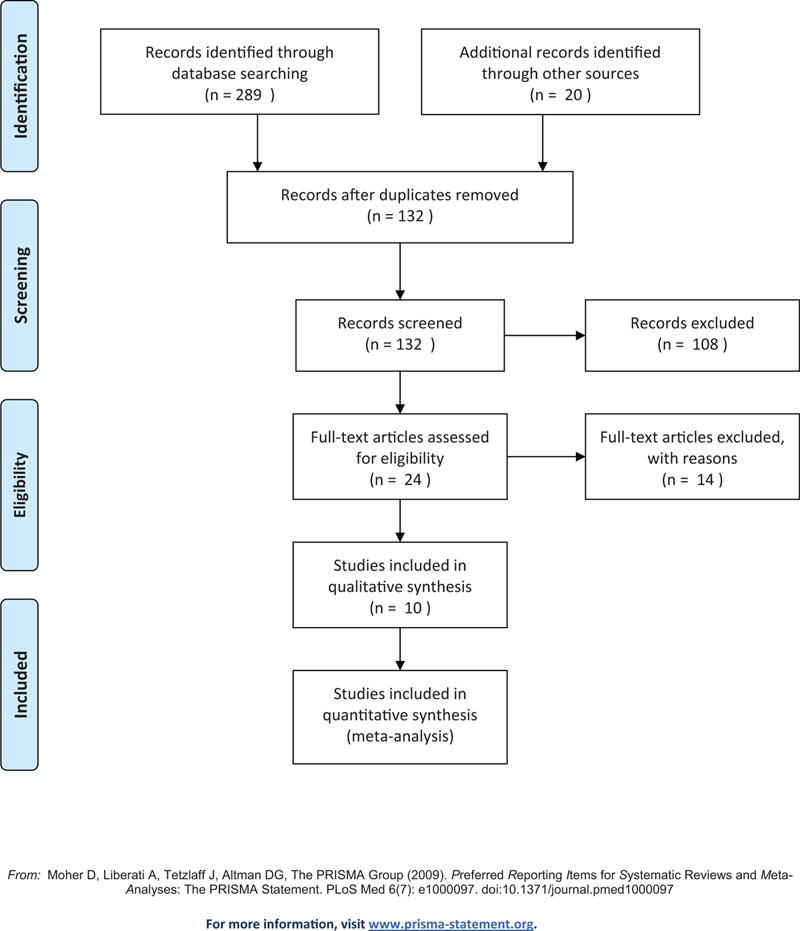
PRISMA flow diagram.

All articles downloaded will be imported into endnoteX9 to remove the identical studies. Two investigators will independently filtrate the title and the abstract of every record to exclude articles inconsistent with the study. The full text of the qualified literature will be investigated and then the authors will extract the data from eligible studies to assess and determine whether the trial meets the inclusion criteria. A third author will be consulted for an expert opinion in the event of any contradiction. The flow chart of the selection process was summarized in Figure 1.

#### 2.4.2 Data extraction

Based on the data extraction form from Cochrane Reviewer's Handbook 5.3.0, 2 investigators will separately extract the following detailed information:

1. General information: title, authors name, journal, publication year, country.
2. Participants: gender, age, duration of disease, pain spots.
3. Interventions: herbs composition, dose, frequency of administration.
4. Outcome: pain score, pain time, adverse events.

#### 2.4.3 Risk of bias assessment

We will assess the methodological quality of randomized controlled trials in accordance with the RCT quality assessment criteria recommended in the Cochrane Reviewer's Handbook 5.3.0. Seven items will be concluded: generation of the random sequence, allocation concealment, blinding of participants and investigator, blinding of outcome assessment, incomplete outcome data, selective reporting, and other biases. The methodological qualities of the included studies will be rated as being “low” (representing a low risk of bias), “high” (for a high risk of bias), or “unclear” (for a medium or unknown risk of bias). Any disagreements between the reviewers will be resolved through discussion or consulting the third reviewer.

#### 2.4.4 Data synthesis

We will use RevMan 5.3 software to perform the meta-analysis. Relative risk (RR) with 95% confidence intervals (CI) will be used for dichotomous variable, and weighted mean difference (WMD), or standard mean difference (SMD) with 95% CI for continuous variable.

#### 2.4.5 Assessment of heterogeneity

Heterogeneity will be evaluated by both Chi-Squared test and *I*^2^ statistic. When *I*^2^ ≥ 50%, *P* < .01, random-effects model will be used, while the other situation is regarded as being indicative of no heterogeneity, and the fixed effects model will be applied. If significant heterogeneity between groups exists, we will explore the reasons from various aspects, such as baseline demographic differences, measurement differences. Necessarily, sensitivity analysis or subgroup analysis would be adopted to explain the heterogeneity.

#### 2.4.6 Subgroup analysis

We will perform a subgroup analysis to explore the potential sources of heterogeneity. This can be discussed as follows:

1. Treatment duration.
2. Course of disease.
3. Functional classification of external drugs.

#### 2.4.7 Sensitivity analysis

Sensitivity analysis will be performed to assess the robustness and reliability of results. By means of changing the inclusion criteria, excluding low-quality studies and using different statistical analysis methods, Changes in RR will be observed. If a literature excluded that has a significant effect on the merged RR, it is considered that this literature is sensitive to the merged RR, otherwise it is not.

#### 2.4.8 Publication bias

When studies included in meta-analysis are more than ten, a funnel plot would be drawn and generated to evaluate the potential publication. Funnel map asymmetry demonstrates publication bias. If necessary, we will put Begger tests into use.

## 3 Discussion

ETCM has a great advantage in the field of surgery and dermatology.^[19]^ Drugs from topical treatment produce systemic effect by passing through cuticle then absorption into bloodstream or nerve reflex stimulation, local anti-inflammatory and analgesic effect by means of acupuncture point and transdermal absorption.^[20,21]^ However, the specific mechanism of ETCM in treating PHN has not been clarified. As guided by the principle of TCM syndrome differentiation, various external treatments have been put into use due to the significant curative effect, in spite of ambiguous mechanism. For example, it is a good choice for applying external agents the medicinal component of which is regulating Qi and activating blood analgesia into the syndrome of qi-stagnation and blood stasis.^[22]^ ETCM, widely used in China, deserve attention and promotion, but its efficacy and safety have not been systematically evaluated.^[23]^ Therefore, we will use systematic review and meta-analysis to evaluate the efficacy and safety of ETCM, expecting that the review could provide more options for the treatment of PHN.

## Author contributions

Tao Lu and Jinzhou Tian conceived of the study; Yize Sun and Zhu Fan searched the literature and performed the data analysis; Biyuan Liu conducted methodological supervision; Zheyi Wang drafted the manuscript; All authors have approved the final manuscript.

**Conceptualization:** Zheyi Wang.

**Data curation:** Zheyi Wang.

**Formal analysis:** Zheyi Wang.

**Funding acquisition:** Tao Lu.

**Investigation:** Yize Sun, Tao Lu.

**Methodology:** Yize Sun.

**Project administration:** Yize Sun.

**Resources:** Yize Sun.

**Software:** Biyuan Liu.

**Supervision:** Biyuan Liu, Zhu Fan.

**Visualization:** Zhu Fan.

**Writing – original draft:** Yize Sun.

**Writing – review & editing:** Jinzhou Tian, Tao Lu.
